# Measuring the Built Environment in Studies of Child Health—A Meta-Narrative Review of Associations

**DOI:** 10.3390/ijerph182010741

**Published:** 2021-10-13

**Authors:** Adriana Ortegon-Sanchez, Rosemary R. C. McEachan, Alexandra Albert, Chris Cartwright, Nicola Christie, Ashley Dhanani, Shahid Islam, Marcella Ucci, Laura Vaughan

**Affiliations:** 1 Centre for Transport Studies, Department of Civil, Environmental and Geomatic Engineering, University College London—UCL, London WC1E 6BT, UK; nicola.christie@ucl.ac.uk; 2 Bradford Institute for Health Research, Bradford Teaching Hospitals NHS Foundation Trust, Bradford BD9 6RJ, UK; rosie.mceachan@bthft.nhs.uk (R.M.); chris.cartwright@bthft.nhs.uk (C.C.); shahid.islam@bthft.nhs.uk (S.I.); 3 Extreme Citizen Science Group, Department of Geography, University College London—UCL, London WC1E 6BT, UK; a.albert@ucl.ac.uk; 4 Space Syntax Laboratory, The Bartlett School of Architecture, University College London—UCL, London WC1H 0QB, UK; ashley.dhanani@ucl.ac.uk; 5 UCL Institute for Environmental Design and Engineering, The Bartlett Faculty of the Built Environment, University College London—UCL, London WC1H 0NN, UK; m.ucci@ucl.ac.uk

**Keywords:** built environment, streets, children, meta-narrative review, non-communicable diseases, health outcomes

## Abstract

Although the built environment (BE) is important for children’s health, there is little consensus about which features are most important due to differences in measurement and outcomes across disciplines. This meta-narrative review was undertaken by a multi-disciplinary team of researchers to summarise ways in which the BE is measured, and how this links to children’s health. A structured search of four databases across the relevant disciplines retrieved 108 relevant references. The most commonly addressed health-related outcomes were active travel, physical activity and play, and obesity. Many studies used objective (GIS and street audits) or standardised subjective (perceived) measurements of the BE. However, there was a wide variety, and sometimes inconsistency, in their definition and use. There were clear associations between the BE and children’s health. Objective physical activity and self-reported active travel, or obesity, were positively associated with higher street connectivity or walkability measures, while self-reported physical activity and play had the strongest association with reduced street connectivity, indicated by quieter, one-way streets. Despite the high heterogeneity found in BE measures and health outcomes, the meta-narrative approach enabled us to identify ten BE categories that are likely to support children’s health and be protective against some non-communicable disease risk factors. Future research should implement consistent BE measures to ensure key features are explored. A systems approach will be particularly relevant for addressing place-based health inequalities, given potential unintended health consequences of making changes to the BE.

## 1. Introduction

The role of the built environment (BE) in shaping health outcomes is widely recognised as important in establishing healthy behaviours in childhood [1]. In the case of preventing non-communicable disease (NCD) by creating opportunities for healthy behaviours, environments that encourage active travel—namely walking or cycling as part of one’s daily routine—or settings that provide access to parks have been shown to reduce obesity and vulnerability to other obesity-related diseases, such as diabetes or coronary heart disease [2]. So-called obesogenic environments have been found to correspond to problematic lifestyle behaviours, including poor diet—for which either a “food desert” or an abundance of fast-food outlets is an added risk factor [3]. Yet, the causal pathways between BE variables (such as dangerous roads or inaccessible footpaths) and health outcomes (such as increased rates of obesity) are yet to be proven [4]. We aimed to overcome the apparent lack of collaborative partnerships that are required to pursue such research by undertaking this review with joint expertise in public health, epidemiology, and social and urban/geographical analysis.

The impacts, positive or negative, that the BE can have on health and wellbeing can potentially be magnified in cities, where high densities can enable walkable and sociable communities but can also lead to overcrowding and exposure to air pollution [5]. Similarly, the positive or negative effects of the BE on health and wellbeing will be amplified in line with the specific needs of the different population groups. For example, the difficulties associated with the need to cross wide streets or roads might be addressed by providing pedestrian crossings. However, crossing distances or crossing times that are estimated for the average walking speed of healthy adults might not be adequate for the walking speeds of other population groups, hence the importance of understanding the BE interaction with people of all conditions and all ages, especially children [6].

The importance of the BE in shaping health outcomes for children is well known, as is the impact of disease in childhood on later life morbidity [7]. Early life and childhood are critical periods where trajectories of health and wellbeing are established that last across the life course [8]. Improving health in childhood thus has the greatest potential for the prevention of later chronic disease [7,9], with the cost savings associated with “preventing” ill health being well established [10]. Over the last decade, various systematic reviews have found that the BE has the potential to support children’s health by promoting increases in the amount of time spent in outdoor play [11], increasing levels of walking whilst reducing pedestrian injuries [12], and enabling active travel to school [13,14], which has been found to lead to increased physical activity and improvements in body weight, cardiovascular fitness, and independent mobility [15]. However, the findings from these reviews have also highlighted that the quality of the studies of the effects of the BE on public health is usually weak because of the use of subjective or non-standardised health outcome measures as well as issues with study design, which are criticised for being opportunistic, non-randomised, or lacking in a follow-up of study participants [13]. It has also been noted that studies in public health tend to be reliant on relatively imprecise or inconsistent descriptions of the BE (such that the measure of “street connectivity”, for example, might refer variously to the number of intersections within an area as the crow flies, or the number of intersections along a route to school) [16] or inconsistent use of spatial concepts such as buffer size [17]. Various reviews have commented that in studies of BE determinants of mental health and physical activity in children and adolescents, the heterogeneity of BE measures is the norm, and have stressed the urgent need for consistency in operational definitions of BE measures [18,19].

The heterogeneity inherent in the measurement of both health outcomes and BE features, including the apparent interchangeable use of objective and subjective measurements, means it is a significant challenge to isolate the key BE features that are linked to children’s health [20]. Even when there is wide recognition of the significance of certain BE attributes, such as street connectivity in promoting health activities, the evidence remains ambiguous [21,22]. This in turn means that there is limited evidence to guide urban planning and policy decisions to shape healthy environments [23]. The aim of this review was to contribute to addressing this challenge by conducting a meta-narrative analysis of the associations of BE attributes and health outcomes measured in studies of child health, focusing on studies that considered objective (e.g., number of intersections within a set walking distance from home) or standardised subjective measures (e.g., a standardised street perception survey) of the BE.

Here, we have undertaken a meta-narrative review, which is a method of systematically reviewing complex topics that have been conceptualised and studied in different ways by different groups of researchers [24]. In this case, a meta-narrative analysis was appropriate as it allowed us to understand how the BE is conceptualised in studies from various disciplines and how it was associated with several health outcomes. By conceptualising BE measures into common narratives or categories, we can, to some extent, overcome the limitations posed by the inconsistency of measures in this field. We also envision that the meta-narrative approach will enable a more comprehensive understanding of the BE and child health interactions so that we can provide some clarification to ambiguous findings from previous studies. Moreover, we focus on objective or standardised subjective (perceptions) measures to attempt to further reduce inconsistencies and biases while gaining insights regarding the actual physical attributes of the BE that can be changed to improve health and wellbeing.

More specifically, the aim of this meta-narrative review was to gain a better understanding of the methods used to study the complex interaction between the BE and children’s physical and mental health and wellbeing. We aimed to focus on studies that explore relationships between BE measures and non-communicable disease (NCD) health outcomes or health activities in children and young people (aged under 18).

## 2. Methods

Our review aimed to ensure that the interdisciplinary nature of our research question would be adequately broad in terms of methods, populations, and measures of studied variables and outcomes. Consequently, a meta-narrative review consistent with RAMESES standards [24] was identified as the most suitable method for analysing the literature and for synthesising the results.

### 2.1. Review Team Characteristics

The authors of this review are part of the Healthy Places stream of the ActEarly consortium, which is investigating the impact of interventions in the built environment (BE) on child health [25]. A.O.-S., L.V., and R.M. were the principal researchers. The remaining authors contributed at key junctures in this year-long exercise. The team were from the following disciplines: public health (R.M. and C.C.), built environment (L.V. and A.D.), transport safety and planning (N.C. and A.O.-S.), citizen science and participatory methods (S.I. and A.A.), and sustainable environmental design (M.U.).

### 2.2. Meta-Narrative Review Principles

To complete this review, the authors followed the six guiding principles of the meta-narrative review method [24] as described below:

1. Pragmatism: the review was guided by the authors’ expertise to define the search concepts considered to be relevant in the association between the BE and child health outcomes to bring about the most useful evidence for public health, transport, and planning researchers and practitioners.
2. Pluralism: the topic of the review was informed by the results of all the studies that fulfilled the search and inclusion criteria, and this resulted in a wide evidence base drawing on several disciplines: architecture and planning, environmental sciences, epidemiology, geography, medicine, psychology, public health and transport, among others.
3. Historicity: the search covered research published in the last ten years to take into account how the topic has been shaped over a large enough time frame that would capture the variety of relevant methods that have emerged in recent years and that have not been captured in similar reviews completed in recent times, while being pragmatic regarding scope (the initial returns from the search were over 2000).
4. Contestation: the review included studies from different disciplines that looked at different health outcomes or activities; this inevitably resulted in several heterogeneous outcomes to be analysed. However, this panoramic view of the associations enabled a deeper analysis of observed conflicting results.
5. Reflexivity: throughout the review stages, the researchers reflected on the findings individually when analysing the data and collectively when reporting and discussing the results.
6. Peer review: the emerging findings were presented to the research programme’s executive group comprising a multidisciplinary team of experts—their feedback guided further analysis.

### 2.3. Information Sources, and Search Strategy

The research team identified three concepts that derived from the research questions to apply to the search: (i) *Streets*, (ii) *Built Environment* (BE), and (iii) *Health*. The *Streets* concept was chosen to ensure that we would capture studies that considered the BE as a measurable, human-scale environment (implementing the principle of pragmatism). The *Health* concept included two sub-concepts: health activities and behaviours, and health outcomes and wellbeing, to capture sufficient breadth of studies that relate to how the urban BE interacts with people’s life (implementing the principle of contestation). The team completed a brief scoping search of the academic and grey literature and identified key studies from each member’s respective fields, from which keywords were added, and appropriate medical subject headings (MeSH) were used to refine the terms used (implementing the principle of pluralism).

**Figure 1:**
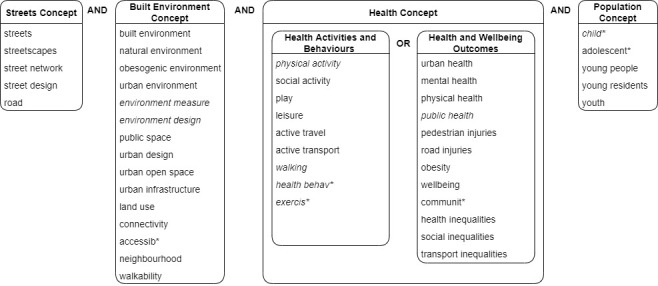
Keywords by concept for the search strategy. (Italics indicate Medical Sub-Heading terms and * was included to capture permutations.)

The lead author then completed trial runs of the search strategy in all databases, testing the results by confirming the retrieval of the key reference papers. Initial results were jointly screened by members of the research team to suggest minor adjustments to the keywords to improve the accuracy of the search. The full list of keywords for the three search concepts was compiled and reviewed by the full research team. *Population* was added at this stage to limit the review to children’s health, with age range set at 18 or younger. We refined the search strategy after consultation with an information specialist. The syntax of indexing terms was adapted for each database’s requirements. Figure 1 presents the final list of search keywords by concept.

To further ensure the implementation of the pluralism principle, the search strategy was completed in four databases to maximise coverage of the relevant disciplines: Medline and Embase from Health and Biomedicine; PsycINFO from Psychology; Scopus from Science and Social Science. No restrictions to (English) language were used to avoid bias but the full-text version of the articles needed to be available to be included. All references were downloaded and imported into reference manager software.

The key inclusion criteria were as follows (see detail in Supplementary Material S1):

Quantitative, objective, or standardised audit, or standardised perceptions questionnaire, measures of physical or spatial aspects of the street; and

- Objective or standardised measures of the urban BE;

and

- Objective, observed, or self-reported measures of physical or social activities such as walking, cycling, active travel, recreation walking or playing OR Objective or self-reported measures of physical or mental health and wellbeing outcomes;

and

- Studies measuring health outcomes and health behaviours in relation to children and young people.

**Figure 2:**
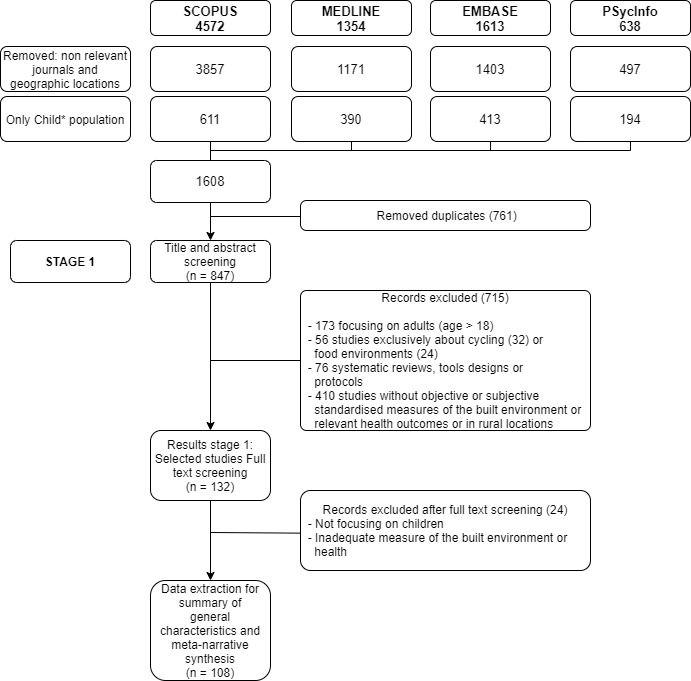
Study flowchart. * was included to capture permutations.

Studies were excluded if they did not involve measurements of the physical BE (e.g., studies that solely considered air pollution or natural environments and greenery) or were reviews, tool designs, or protocols. Similarly, studies exclusively about cycling or food environments were excluded. Although these two topics are highly important for the health of children, and particularly problematic in deprived urban areas, we considered that the topics have a degree of complexity that would deserve a review in their own right. Moreover, some BE attributes that promote cycling can have an opposite effect on walking [26]. Finally, given that the BE may not be comparable across countries with varying income levels, we limited our review to evidence from upper-middle and high-income countries, using the World Bank classification (available from http://data.worldbank.org/about/country-classifications),(accessed on 26 February 2021). Figure 2 presents the study flowchart with the details of total number of studies retrieved, screened, and analysed.

### 2.4. Evaluation and Coding for Title, Abstract, and Keywords

Of the 857 records resulting from the initial searches across four databases, 749 studies were excluded: either because they were out of scope (56), or did not include children (173), or were reviews or protocols (76), did not include objective or standardised measures of the BE (410), or were duplicates (34). All records were double-coded for inclusion by A.O.-S. and L.V.; a random sample of records (*n* = 200) was double-coded for inclusion by all the authors. Inter-rater reliability and agreement were estimated using k Fleiss, a statistic measure for multiple coders and nominal classifications [27,28]. Good inter-rater agreement was obtained (kappa = 0.85). Disagreements were discussed and resolved.

## 3. Study Characteristics

The first step of the review consisted of building a broad description of the characteristics of the 108 selected studies. To do this, the full text of these 108 studies was read by two researchers (A.O.-S. and L.V.). When conducting this revision, the researchers extracted data across six pre-defined dimensions: geographic location; study design, sample size, and age; social factors; study area; built environment measures; and health activities/behaviours and health outcome measurements and tools. Section 3.1 to Section 3.6 present the characteristics of the 108 studies for each of the six dimensions and Table 1 presents the summary of study characteristics.

### 3.1. Geographical Location

The majority of studies reviewed were conducted in the USA (33%), Europe and the UK (27%), Canada (15%), and Australia and New Zealand (13%). The remainder were located in Central (5%) and East Asia (3%), Latin America (3%), and the Middle East (2%).

### 3.2. Study Design and Sample Size

The majority of studies (79%) were cross-sectional. Thirteen studies (12%) were longitudinal (six of which were longitudinal cohort studies). Seven studies (6%) used other study designs: two descriptive studies, one qualitative study (focus groups and semi-structured interviews), and one pilot non-randomised controlled evaluation study, all of which used samples of less than 100 participants. The remaining studies were one participatory study and two pre/post-test studies (all with samples between 101 and 500). The sample sizes ranged from 11 to 326,383. Four studies also included parents/carers in their sample. One study used a sample of 124 schools without mentioning the number of pupils. Three age categories were identified as broadly comparable across the studies: Early years (0–4 years old) in three studies; Children (5–11 years old) in 27 studies; and Adolescents (12–18 years old) in 32 studies. Forty-six studies bridged across Children and Adolescent groups.

### 3.3. Social Factors

Most studies controlled for children’s age, sex, and ethnicity. Twenty-four studies considered measures of socio-economic status (SES) either for the area or neighbourhood or for the household. Parental education level was commonly used as a proxy for the household SES. Eleven studies used SES or walkability levels or other similar measures to determine neighbourhood sampling. For this purpose, each area or neighbourhood was classified as high or low for SES and high or low for walkability. This classification enabled the comparability of the areas within the research location, which could be used for identifying control areas or as a control variable for statistical analysis. Some studies included other socio-economic variables such as the number of vehicles per licensed driver in the household or qualification for free or reduced-price meals (typically this was in US studies). These variables were used to adjust for deprivation and/or race and ethnicity.

### 3.4. Study Area

No consensus or standardised approach for the definition of the study area—normally an area around participants’ home, school, or their route to/from school—was identified; however, many studies defined the distance used as an *acceptable walking distance threshold*. This threshold was determined as a buffer area that could be defined based on one, or various, specific distances. The studies ranged in their focus or scope, as described in Table 1. The distance used to define the buffer presented a wide variation, though 800 m was the most often used (21, or 19% of all studies) as it approximates a 10-min walk (assuming an average walking speed of 5 km/h), a common standard of walkable neighbourhoods [29,30,31].

### 3.5. Built Environment (BE) Measures

Two main types of BE measures were identified: *objective* measures, which included those gathered using GIS methods or via standardised street audits, collected by trained researchers; and *standardised subjective* (perceived) measures reported by participants via street perception questionnaires. Most studies used objective measures either alone (76) or in combination with street perceptions (29). Table 1 provides detail on this.

The most common variables are either an aggregate walkability measure (namely land-use mix; intersection density, street connectivity, or street density; residential density; and transit or public transport) or one or more of the individual parameters that comprise walkability measures. Many studies used only one of the individual measures (17). A sub-set of those classified as *street connectivity* (7 of the 62) used space syntax analysis, a validated mathematical method for predicting pedestrian rates based on the configuration of a street network. We defined studies as using *walkability* as a tool only when it was explicitly mentioned in the text.

Other objective measurements used are listed in Table 1. The precise numbers should be regarded with caution—frequently there is a lack of specificity regarding the nature of the data gathered, so distance to a physical activity-enabling destination might be labelled access to land-use in a comparable study (and thus would fall into the table above).

In addition to street level walkability-related measures, 30 studies used street audits, namely direct observation by trained researchers on foot or via the windshield of a car, to document attributes of the street, such as physical incivilities (e.g., the presence of rubbish or graffiti) and the availability of social spaces (e.g., the proportion of porches on houses).

Of the studies that used street audits or perception questionnaires, several used standardised instruments, as presented in Table 2. One example of these is PARA, which uses in-person audits to document features and incivilities at physical activity facilities (i.e., churches, commercial facilities, trails, parks, and schools) [32]. A sub-set of the standardised instruments was adapted for a child-centred environment, such as NEWS-Y [33,34,35,36,37,38] and NDAI-C [39].

### 3.6. Health Activities, Behaviours and Outcomes, Measurements and Tools

Sixty-six studies (61%) measured exclusively health activities. Twenty studies (19%) included only measures of health outcomes and 21 (19%) studies included measures of both activities and outcomes.

Of those that measured health outcomes, the majority used either measured or reported Body Mass Index (BMI). The remaining measures were birth weight, child pedestrian crashes, depression, mental health, positive child development, reported school performance, reported child pedestrian crashes, and reported physical activity injuries occurring in the street.

The dominance of body weight as a health outcome measure indicates the prevalence of obesity-related studies in the literature of the associations between the BE and health in children. In most of these studies, physical activity is included as the intervening factor between the BE characteristics and BMI measures. The limited number of studies (*n* = 4) that considered mental or psychological outcomes in relation to street-level BE features is also noteworthy. Four methods of capturing health activities and behaviours were identified: Objective, Observed, Parent-reported (commonly used for children), and Self-reported (mostly used in adolescents). Of the 88 studies that considered one or more measures of health activities, 36 considered measures related to travel and 44 studies considered measures of physical activity. Four studies used observations of PA, or street or park use. Parent-reported active travel and PA was considered in six studies and parent-reported play in three studies. In general, the largest group of studies (47) considered reported measures, of which 12 used standard tools to capture physical activity or active travel. These included tools to measure self-reported physical activity, as can be seen in Table 2. The multiplicity of measurement tools is one of the features that makes comparison across studies, and indeed opportunities to replicate studies, more challenging.

As described in Table 1, the characteristics of these studies indicate a relative dominance of self- and parent-reported measures for health activities in the reviewed studies. However, the use of accelerometers to gather objective measures of physical activity, active travel, and physical inactivity was also significant. The tendency towards using more than one health activity measure and combining objective and reported measures is also highly relevant.

## 4. Results: Associations between Built Environment and Health Outcomes

In this section, we summarise the main associations between attributes of the BE and children’s health (activities and outcomes) reported in the 108 studies reviewed. All the studies analysed for the summary of associations had samples larger than 100 participants, and 14% of them were longitudinal studies. As described in the previous section, the reviewed studies considered more than 20 different health outcomes; therefore, for this summary, the outcomes are grouped under three categories that mediate health outcomes and a fourth health outcome in its own right: Physical Activity and Play, Physical Inactivity, Active Travel, and Obesity and other health outcomes. The following four subsections report narratively on the associations for each of these four health outcome categories. The two tables at the end of this section present a summary of the total number of studies that found associations for each pair of BE health mediator or outcome.

### 4.1. Built Environment (BE) Correlates of Physical Activity

#### 4.1.1. Physical Activity (PA)

Objective PA was found to be positively associated with intersection density, as a measure of street connectivity [34,35,40,41,42], residential density [34,40,41,43], household density [44] and population density [45], perceived land-use diversity [34,46] and objective land-use mix—in studies for children in early years [47], and walkability (defined as a combination of street connectivity, land-use mix, and residential density measures) [35,40,48,49].

Objective PA was also found to be associated with BE attributes at the street level such as streetscapes supportive of active travel [39,46], street lighting [32], street aesthetics [34,46,50], speed bumps—for boys [51], objective traffic safety—for girls [41], and parents perceived personal safety for all children [52].

Objective PA was found to be linked to accessibility [42,46] and objective [41] and perceived [53] proximity to activity (play/sports/recreation) promoting destinations. In line with this, objective PA was also found to be positively associated with the availability of public open spaces [40], social spaces [54], and parks for adolescents [34,45] and children [41] and other green or blue infrastructure [50], including side trees in the home street [47]. Access to the beach for adolescents [32] and perceived access to green areas [52] were also associated with objective PA.

Self-reported PA in adolescents was linked to perceived personal safety [37], perceived traffic safety [36,55], and perceived street aesthetics [36,53].

Objective PA [41,56] and parent-reported PA [57] were associated with reduced street connectivity, indicated by the presence of dead-end-roads/cul-de-sacs or one-way roads. This is likely to be a result of the relationship of this type of road design with increased traffic safety. Additionally, in the unexpected direction, objective PA and self-reported PA were found to be associated with reduced land-use diversity [35,42,58], and increased PA on non-school days was found to be moderately associated with less walkability [50]. No association between physical activity and walkability was found in two studies [44,59].

Reductions in reported PA were found to be associated with increases in intersection density [58], road connectivity [60], and street connectivity [37,53,61,62]. More high-speed or main roads in the area were linked to decreases in objective PA for children [39], girls [41], and adolescents [45]. Finally, regarding the social environment, parental support for PA [54], going out to walk the dog for adolescents [35], having a supportive social neighbourhood environment for boys [41], seeing other siblings perform physical activity for boys, or parents for girls [56] were positively associated with objective MVPA, and going out with friends was found to be associated with reductions in objectively measured physical inactivity [63].

#### 4.1.2. Parent-Reported Play or Park Use

Parent-reported play or park use was found to be associated with the presence of walking facilities [44], street quality [64], the presence of pedestrian amenities with fewer path obstructions [65], and pavement availability, which can serve as informal play areas [66]. Similarly, parent-reported outdoor play or park use was positively associated with the presence of traffic calming features such as pedestrian crossings with and without traffic lights [66], parental perceived traffic safety [67], perceived personal safety [53,68], and, in the unexpected direction, with less walkability for children in Mexico [65].

Reductions in parent-reported play were associated with increased intersection density [67,68].

### 4.2. Increased Sedentary Time (ST) or Physical Inactivity

Increased sedentary time was associated with less walkability [69] and an increased number of main roads in the area [70]. More objective physical inactivity [70,71] was related to reductions in perceived personal safety. In the unexpected direction, physical inactivity was found to be associated with land-use diversity [33,63,72]. Girls were more likely to be 75% to 85% inactive at age 8–10 and 10–12, respectively [63], and when physical activity decreased, on average, young girls reported the greatest decline.

### 4.3. Built Environment (BE) Correlates of Travel (Including Objective and Self-Reported Active Travel to School and Parent and Self-Reported Travel for Other Purposes)

#### 4.3.1. Active Travel to School

Self-reported active travel to school (ATS) was found to be positively associated with objective and perceived measures of street connectivity. Objective measures include intersection density [55,73,74,75,76,77], space syntax measure of global choice [78], and the presence of a grid-patterned road network [79]. Perceived measures refer to perceptions of street connectivity [38] and the perception of many paths to walk in the home neighbourhood [80]. Objective ATS [26,81,82] was also found to be associated with street connectivity.

Other BE attributes reported as significantly positively associated with self-reported ATS include residential density [38,74,76,77,79], land-use mix in adolescents [73], girls [77], and children [55,83], ground floor attractions and retail density [84], the presence of food outlets [74], and walkability [49,77,80,85,86]. Similarly, walking infrastructure attributes such as proximity to walking paths [87], sidewalk width [84], and streetscapes for active travel [86] were reported as associated with self-reported ATS. Proximity to school was found to be the main variable associated with self-reported ATS for children and adolescents [73,74,76,78,85,88,89] and with objective ATS [26,81,90]. Likewise, objective and reported ATS were associated with accessibility to activity-promoting destinations [55,75,91] and the availability of open public spaces [77,86], greenery [92] [89], greenways [90], and street trees [93]. Some studies found that boys were more likely to walk to school, responding to parental perceptions [89], or that male teenagers were more likely to walk than females of the same age [88].

Self-reported ATS was found to be associated with various perceptions of safety, including personal safety perceptions of adolescents and children [76,79] and parents [87] as well as traffic safety perceptions of children [55] and parents [38]. Objective ATS was found to be associated with observed safety [90].

Self-reported ATS was found to be negatively associated with other traffic-related measurements such as objective measures of traffic levels [89] and speeding traffic [80]. At the individual level, having access to a motorised vehicle at home [76] and travelling by motorised transport [84] were linked to a reduction in self-reported ATS.

#### 4.3.2. Non-School Active Travel

Parent and self-reported active travel were found to be associated with street connectivity [39,43,46,51,91,93,94] and street walking infrastructure such as streetscapes [46] and total length of walking tracks [51].

Social support from peers or family (Hwang et al. 2017), measured as the number of siblings for adolescents [26], the mother’s confidence in the child’s ability [87], or parents’ decision to allow children to play in the neighbourhood, was positively associated with active travel. Parental accompaniment when travelling was positively associated with parent-reported active travel [91]. Self-efficacy [76] and journey enjoyment and satisfaction were positively associated with self-reported active travel [55,86,87].

### 4.4. Built Environment (BE) Correlates of Obesity and Other Health Outcomes

As presented in Table 1, only 39 of the reviewed studies considered health outcomes and, of these, 30 used objective or subjective BMI measures and five studies looked at other health outcomes such as mental health [95], child development, depression, birth weight, and asthma. In line with that, the majority of reported associations are for BMI measures. More precisely, reductions in objective BMI were found to be associated with intersection density [96,97,98,99], walkability [100,101], walking infrastructure [99,102], availability of parks and public open spaces [47,99], accessibility of play and sports destinations [103] and convenience stores [102], and improved traffic safety [47,104].

Objective BMI was found to be positively associated with access to food outlets and the scale of the floor level retail sector [103,105], crime and physical incivilities [106], and the density of bus stops [98] and subway stops [47].

For other health outcomes, such as asthma, mental health, depression, child development, or birthweight, the association with BE attributes was minimal and in several cases in the unexpected direction. However, it is worth noting that within the reviewed references most of these health outcomes were included in only single studies. Exposure to greenery was the one “built” environment feature positively associated with various health outcomes, including birthweight, lower odds of social vulnerability, and, indirectly via its restorative effect, with mental health.

The following two tables present the total number of associations between the BE and physical activity and physical inactivity (Table 3) and the BE correlates of travel and obesity (Table 4). A subclassification for the direction of the association is included: Positive, indicated by (▲), Negative (▼), and No association (◂▸). Only outcomes that were reported in more than three studies are included in the tables. The number in each cell indicates the associations reported for the specific BE–outcome pair. The superscript numbers are the individual level studies that reported the associations. For Physical Activity in Table 3 and Active Travel in Table 4, the columns Total PA and Total Active Travel, respectively, present the overarching summary of the various outcome categories. BE attributes in bold indicate those that have four or more positive associations, and the row colouring indicates the identified emerging categories of BE attributes. See Supplementary Materials for full details of the studies.

## 5. Discussion

The aim of this meta-narrative review was to understand the methods used to study the complex interaction between the built environment (BE) and children’s physical and mental health. Furthermore, we aimed to identify how the BE is defined and measured and the potential effects that specific BE attributes can have on children’s health.

As noted in previous reviews, we found that the operational definition for the built environment (BE) variables in the reviewed studies presented a low degree of conformity [18]. Likewise, the definition of health and activity outcomes varied widely. However, when assessing the interaction between the BE and children’s health, the summary of associations enabled us to identify broad categories of BE attributes that are likely to be protective factors supporting health activities and outcomes such as physical activity, play and active travel, and BMI in children and adolescents. Table 3 and Table 4 in the previous section list the ten broad categories of BE attributes and their association with the studied outcomes. The ten categories include measurements relative to: (i) residential or population density; (ii) intersection density (or other measures of street connectivity); (iii) land-use diversity; (iv) walkability (a composite measurement including the previous three attributes); (v) street-level walking infrastructure and perceptions of street environments; (vi) accessibility or proximity to recreation, sports, or play spaces or facilities, and proximity to school as a the key determinant for active travel to school; (vii) availability and accessibility to public open and social spaces and natural environments such as parks, green spaces, street greenery, and water bodies; (viii) perceptions of safety from traffic and crime; (ix) motorised traffic levels and the presence of main roads; and (x) social support and psychosocial factors.

**Figure 3:**
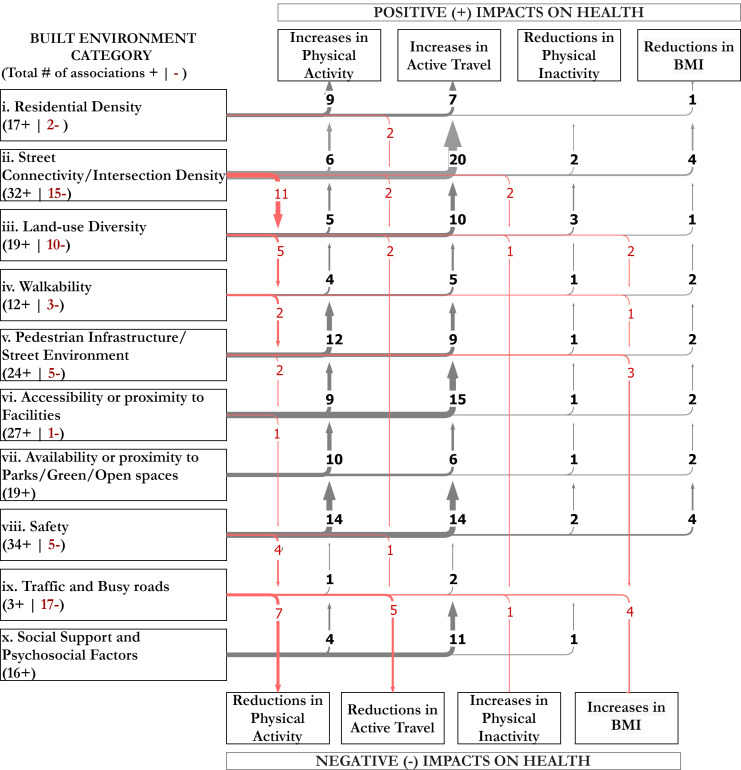
Built environment categories and interaction effect on health mediators and health outcomes. The arrow's width and numbering indicate the number of studies that found the specific association. The number in the brackets under each BE category indicate the total number of studies that found associations between the BE attribute and positive (+) or negative (−) health outcomes.

When examining the detail of the frequency of associations as a proxy measure of the effect of specific BE environment attributes on children’s health, we found that the categories that emerged as most frequently being associated with positive impacts on health—defined by the combined effect on increases in physical activity or active travel and reductions in physical inactivity or BMI—were Safety (34), Street Connectivity (32), Accessibility or proximity to facilities (27), and Pedestrian infrastructure and Street Environments (24) (see Figure 3). Similarly, Accessibility or proximity to parks or open spaces (19), Land-use diversity (19), and Residential density (17) were also found to be frequently associated with positive impacts on health.

We found ambiguity in the effects that BE attributes such as street connectivity and land-use diversity have on health outcomes. For example, the positive association between street connectivity and active travel was striking (20 studies reported this association), while the association between street connectivity and physical activity was also apparent (17 studies), but with high ambiguity in the direction of the associations and higher values (11 studies) for the associations with reductions in physical activity. Finally, we found that the category related to motorised traffic and the presence of busy roads emerged as the most frequently associated with negative impacts on health (17 studies), including reductions in physical activity and active travel and increases in physical inactivity and BMI.

Beyond the identification of the broad BE categories, the analysis of the myriad of measures used to capture the BE characteristics (presented in Section 3.2) and their reported associations with health activities and outcomes allowed us to identify an underlying rationale for selecting BE measures to include in studies of child health. The rationale appears to be the need to capture and quantify the BE environment attributes that act as enablers or those that represent barriers for walking and physical activity. As such, we identified that the ambiguity in the effect of certain BE attributes on children’s health can be better understood in the context of enablers and barriers for the different health activities, such as active travel, physical activity, and play. This analysis of enablers and barriers also indicated that the BE categories are not necessarily independent but that they interact, which may produce a cumulative impact on health outcomes. For example, attributes such as street connectivity and land-use diversity can determine, to some extent, other attributes such as traffic levels, which in turn can also interact with attributes such as perceptions of traffic safety and street environment quality. However, the relationships between these categories are complex and context-dependent, with preferences for BE features likely to vary in different population groups. For example, our review found in one context higher street connectivity to be related to active travel, possibly because it facilitates shorter distances from “A” to “B”, whilst in another study lower street connectivity as assessed by one-way roads or cul-de-sacs was associated with greater physical activity in children. The following are the details emerging from the reviewed literature that provide further clarification on the role of each studied BE attribute as an enabler or barrier for walking, physical activities, or health outcomes such as BMI:

- **Residential density** (which in some cases measures child population density) was found to be an enabler mostly positively associated with both physical activity and active travel—linked to the presence of more people who can complete activities in the area or who can move around to connect with other people.
- **Land-use diversity** (mostly measured as a mix of different types of uses) was considered a physical activity enabler, namely as opportunities to move around or potentially as a proxy for characteristics of street vitality and safety. It was commonly related to active facades and “eyes on the street”, which urban design research has found to provide protection against crime (and fear of crime). Similarly, the presence of uses such as food and retail outlets or convenience stores was identified as a potential reason to engage in active travel. Conversely, in some contexts, mixed land-use was also considered as a proxy for overcrowding and potentially increased traffic, which create a less safe and pleasant environment, thus unsupportive to children’s physical activity. Land-use diversity was also considered a measure of risk for increased BMI, especially when it signalled increased exposure to fast food outlets.
- **Street connectivity** (measured as intersection density, or type, or street linearity, or block size) was generally identified as an enabler for active travel (walking) to school and objective physical activity, as greater connectivity normally leads to shorter routes from a to b. However, many other studies, as with land-use measures, identified increased street connectivity as a deterrent to child-reported physical activity or parent-reported play. This is because more connectivity—especially when measured as intersection density or the number of three- or four-way intersections—is likely to lead to an increase in the speed and volume of motorised traffic. In line with this, reduced street connectivity, which results in reductions in traffic levels and speeds, was seen as an enabler for objective and self-reported physical activity. This evidence suggests that increases in street connectivity can be considered proxy measures for reductions in traffic safety, in which case, reducing through traffic (e.g., via designed cul-de-sacs or planned school streets) creates “quiet ways”, which improve the perception of traffic safety and are therefore seen as better suited for encouraging physical activity and play [107].
- **Walkability** (namely the composite index) was mostly found to be an enabler of walking for active travel.
- **Walking infrastructure** and aesthetics assessed at the street level—via street audits or street perception questionnaires—were identified as enablers of active travel, physical activity, and play.
- **Availability and proximity to green and blue infrastructure** emerged as key enablers mainly of physical activity and play. **Access to parks and open spaces** was identified as one of the most protective environmental factor for children’s physical activity [40]. For travel, proximity to schools was the strongest enabler for active travel to school, in line with previous research [89].
- As in previous studies, **positive perceptions of traffic and personal safety** in the area (by both children and parents) were found to be mostly enablers for physical activity and active travel.
- **Public transport or transit accessibility**, which can be seen as a proxy for access to places beyond the neighbourhood—so generally expected to be an enabler—was measured in 20% of our reviewed studies, but with only three reported associations, two as barriers to children’s physical activity and one as an enabler of active travel. This may indicate that public transport accessibility, usually measured as the density of train stations or bus stops, can be, as land-use diversity, a proxy for high levels of street activity that lead to barriers to physical activity such as overcrowding and increased traffic.

In line with previous research [108,109], our review underscored the relevance of psychosocial factors (e.g., perception of physical self-efficacy, social support from peers and family or social norms and enjoyment and satisfaction with active travel to school (ATS) and for other purposes).

Regarding health outcomes, we found that the same BE attributes that enabled physical activity and travel were associated with reductions in objective BMI. This might be partly because physical activity is an intervening factor between the BE and obesity [96]. We also found exposure to greenery as the only BE attribute associated with the various health outcomes. This finding supports results from previous studies in adults that highlight the positive effects of exposure to greenery on health and wellbeing directly, and indirectly through routes such as opportunities for physical activity, social contact, stress reduction, and attention restoration [110,111]. Correspondingly, the findings highlight the value of streetscapes, street art, and street-side greenery as attributes that promote health and wellbeing through soft engagement that leads to attention restoration.

Finally, regarding sex differences, we found that some studies segmented results by sex to assess any observed differences, with some finding that girls had lower levels of physical activity or were more affected by the conditions of the environment and that boys or male teenagers were more likely to walk to school. However, due to the diversity of measures of physical activity and active travel, and the inconsistency of age ranges used in the studies, it is difficult to draw any general conclusions in this regard.

The findings discussed in this section suggest that researchers should limit the desire to find universal associations between BE variables and health outcomes, and instead, consider the specific environmental and social context within which they are working. In fact, as described in the results section, it is possible that the same BE attribute can have the complete opposite effect on different activities or populations. For example, street connectivity measured as intersection density was found to increase objective PA and objective active travel to school but decrease self-reported PA and parent-reported play. Hence, our findings expand existing knowledge by bringing clarity to the apparent contradictory effects of the BE on children’s health by showing that the nature of the interactions appears to be, to a great extent, conditional upon the type of activity taking place and other elements of the context [107] or characteristics of the individual [108]. Moreover, these contextual characteristics will determine if a specific BE attribute acts as an enabler or a barrier for health-promoting behaviours.

## 6. Implications

### 6.1. For Research

We found a wide variation in the way the built environment (BE) was assessed. As highlighted in previous studies [18,112], the lack of a standard validated set of BE criteria important for health means that when trying to characterise the BE we faced a large list of potential indicators, which made comparability and synthesis of study findings extremely difficult.

Given that almost all the BE measures were found to be enablers or barriers for positive, and in other cases, negative health behaviours, the direction of their association with physical activity and health presented equivocal results, as shown in Table 3 and Table 4and Figure 3. This lack of consistency also applies to the myriad of BE and health activity tools and instruments. Added to this are other difficulties that stem from the use of human observers to assess neighbourhoods for their environmental qualities, especially in the case of observed disorder, which can involve inherent biases in the assessment; seeing disorder is “imbued with social meanings” that cannot be divorced from an observer’s individual perception [113].

Also notable are the relatively few instances of walkability indexes, or street audits, or definitions of environmental areas, which are adapted to be specific to the needs of children, with the Neighbourhood Destination Accessibility for children as one of the few exceptions. This is important not only because of the differences in how children perceive their environments, but also because of how they use them differently. For example, previous studies have found that children explore their environments differently when accompanied by adults than when walking independently, with young children also having various types of meandering movement [114].

The lack of consistency of defining the effective study area has been noted before, which indicated that studies in this domain rarely use detailed GIS databases and thus are reliant on approximations of urban form measures such as the distance between home and school [26]. There are other differences to be noted in this context between studies that look at home environment, many that look at access to park facilities, and those that are about school, or route-to-school environments. Indeed, the latter type of study was the dominant approach found in our review, rather than free walking in the area. This may be a gap that could be filled in the future.

To address some of these consistency issues [18], researchers have suggested that the grouping of the BE variables in categories—such as population, built form, land-use, road/street environment and pedestrian infrastructure, facilities and amenities, neighbourhood green and open space, and composite measures (e.g., walkability)—could be an initial step towards establishing consensus regarding which BE attributes are important to measure in studies of child health [18]. Other BE categories that are used with some level of consistency in the transport planning literature—and were found in four instances in this review—are the 5D variables, comprising Density (intensity of use per area unit), Diversity (mix of use), Destination accessibility (access to activities and services, including public transport), Distance (to facilities and public transport), and Design (street network or street environment) [115,116,117,118]. However, these variables focus on the type of measurement more than BE attributes as such, so do not provide a useful alternative for studies in this domain.

In contrast, the ten categories that emerged from our review are, to some extent, a combination of the previous two sets, and also incorporate perception measures. The categories and examples of common measures for each category are presented in Table 5.

Hence, greater consistency could be achieved by acknowledging certain categories, as identified in this and previous studies, and framing the variables to be considered within a study of those categories. Similarly, research in this topic area would benefit from the use of certain standard operational definitions for key measures. For example, the use of standard definitions of walkability [119,120] could be valuable for comparability when characterising the BE in studies of active travel. Likewise, the use of analytical tools that capture the effect of spatial systems on human behaviour in a more comprehensive matter could be valuable for assessing the street environment’s potential to be used for walking or physical activity. Approaches such as space syntax analysis that take into account the shape, geometry, and configuration of the street environment could provide insights in this direction, as has been found elsewhere [119].

A final remark regarding objective and subjective (perceptions) measures of the BE is that they are both important and should be included in studies of BE for child health, but they need to be disentangled and may not always be used interchangeably. This is because, even if the objective and subjective measures correspond to the same BE attribute, in many cases they are capturing different aspects of it. The subjective measures considered in this review captured, through standardised questionnaires, people’s perceptions of the objective BE. However, when people are asked about an objective BE attribute such as density, they do not think in objective terms (e.g., population per square metre), they think in their experience of that density (e.g., crowding) [121]. Therefore, these subjective measures do not necessarily hold a direct relation with the objective accounts as they are framed by each person’s preferences, and indeed their life experience, but, nonetheless, they are expected to be better predictors of some behaviours [122] or subjective wellbeing [121]. Despite this, subjective measures have also been considered problematic because of them being prone to recall error, same-source bias, and difficulty to translate into practice [123]. In sum, both measures provide valuable insights for the delivery of health-supportive BE. Subjective measures give insights into children’s experience when interacting with the BE, which links their wellbeing and health with the BE. Objective measures, on the other hand, give practitioners the guidance they need to identify the physical elements that can be changed to promote the desired positive health outcomes.

### 6.2. For Practice and Policy

Our review highlighted the many complexities inherent in understanding how the BE impacts on children’s health and how these vary depending on aspects of the context such as the activities taking place.

For **optional** activities that provide reasons to be out and about in one’s neighbourhood, the role of the BE is to provide pleasant and safe environments, such as high-quality footways and clean and green street environments. Similarly, land-use diversity or proximity to recreation sites can incentivise active travel to visit these destinations. Yet, there can be barriers to such provision, so, for example, a planned local centre that has the positive outcome of creating an attractive mix of activities for people living and working in the vicinity might lead to overcrowded and unpleasant environments and traffic congestion, especially in cultures with high use of cars for mobility.

For **necessary** activities such as travel to school, well-connected footways and proximity to destinations are the most effective means. Yet, there are several potential barriers, varying between low perceptions of safety from traffic and crime, problematic traffic levels and speeds (resulting from the presence of main or busy roads), or a lack of adequate crossings.

In sum, this review underscores that the provision of a health-supportive BE for children requires a systems approach that enables an understanding of the wider context. This systems approach puts policy and decision-makers in a better position to assess competing priorities, identify BE enablers and barriers for children’s health, and mitigate potential unintended consequences.

## 7. Strengths and Limitations

Our multi-disciplinary, structured process has been beneficial in ensuring that the reading of research in this inherently complex domain is sufficiently broad. Yet, we acknowledge that our focus on approaches for measuring the built environment (BE) that used objective and standardised subjective (perceptions) measures at the area and street level may have not accounted for other approaches to studying the relationship between child health and the BE (e.g., studies that only considered measures at the area level such as those looking at spatial distributions of traffic collisions, or amount of green spaces in an area). Ultimately, the lack of consistency in measurements of the BE has meant it is difficult to compare across studies, so our reporting of quantitative results is somewhat constrained. However, the meta-narrative approach used in this review, with its ability to study concepts with heterogeneous definitions, provided the tools to synthesise the data in an insightful manner and draw valuable conclusions.

## 8. Conclusions

Our review found clear evidence on relationships between some BE indicators and children’s health. In particular, our study provides strong evidence for the need to shape a health-supportive built environment (BE) for children that encourages walking and physical activity while reducing the potential barriers (whether physical or mental) for doing so.

Our review’s focus on objective and standardised subjective methods of capturing BE characteristics has allowed us to gain new insights into how research in this domain might benefit from greater consistency in measuring the BE in health research. It is recommended that future studies include a minimum set of BE indicators across a range of categories to aid comparability and knowledge generation. Our review also highlighted specific gaps in the knowledge regarding children, their environments, and health outcomes, especially regarding research in the health of children living in deprived neighbourhoods. It is notable that we found few studies with a mental health focus (other than a handful that considered exposure to parks or greenery), given that this has been recently identified as a critical gap both in health and in urban design practice, requiring “a radical change in attitude” [124].

Finally, the evidence gathered in this review stressed the fact that in the absence of strategies to reduce traffic, street improvements that lead to significant increases in children on the streets are likely to result in increased risk exposure (i.e., to air pollution, noise, or traffic collisions). This risk exposure can offset the positive health effects of street-based physical activity and active travel by affecting physical health directly (with increases in child pedestrian injuries or respiratory illness) and mental health indirectly by worsening the travel experience, creating stress and attention fatigue [125].

We therefore argue that a systems approach is vital in this context to ensure that improvements to the BE do not conflict with the aims of providing a health-supportive environment. Using a systems approach when planning improvements of street environments will help to ensure that any associated barriers, or risk exposures, are simultaneously reduced. Such an approach is especially relevant when addressing place-based health inequalities, bearing in mind that highly deprived areas are likely to have the least favourable health-related BEs.

## Figures and Tables

**Table 1:** Characteristics of selected studies (*n* = 108).

Characteristics	No/% Articles		Characteristics	No/% Articles	
**Geographical Region**			**Built Environment Measures**		
Australia and New Zealand	14	13%	Street connectivity/intersection density	62	57%
Canada	16	15%	Land-use diversity	45	42%
Central Asia	5	5%	Residential density	41	38%
East Asia	3	3%	Public transport accessibility	20	19%
Europe and UK	29	27%	Walkability	28	26%
Latin America	3	3%	Street audit	30	28%
Middle East	2	2%	Distance to PA destinations	24	22%
US	36	33%	Distance to school	22	20%
**Study Design**			Traffic calming measures	12	11%
Cross-sectional	85	79%	Traffic levels	12	11%
Longitudinal	13	12%	Crime levels	9	8%
Other	10	9%	Greenness and/or vegetation or tree cover	5	5%
**Sample Size**			**Health Activities and Behaviours**		
<100	3	3%	Observed MVPA/PA (accelerometer)	29	27%
100–500	38	35%	Self-Reported MVPA/PA	11	10%
501–1000	20	19%	Observed ST/PIA (accelerometer)	3	3%
1001–3000	25	23%	Self-Reported ST/PIA	1	1%
3001–10,000	12	11%	Observed PA/street or park use	4	4%
>10,000	6	6%	Parent-Reported Play	3	3%
**Age**			Parent-Reported Active Travel/PA	6	6%
Adolescents (12–18 years old)	32	30%	Observed Active Travel to School	2	2%
Children (5–11 years old)	27	25%	Self-Reported Active Travel to School	10	9%
Children and Adolescents (5–18 years old)	46	43%	Self-Reported travel to school (trips, mode)	12	11%
Early years (0–4 years old)	3	3%	Observed travel (mode, route)	3	3%
**Study Area (Size)**			Self-Reported active travel non-school	2	2%
100 m–2 km (mode 800 m, *n* = 21)	55	51%	Self-Reported travel (trips, mode, route)	1	1%
2 km–5 km	53	49%	Self-Reported energy intake and expenditure	1	1%
**Study Area (Definition)**			No health activity measure	20	19%
Home and school (Euclidian)	4	4%	**Health Outcome**		
Home and school (network)	3	3%	No health outcome	66	61%
Home (Euclidian)	27	25%	Asthma-related	2	2%
Home (network)	11	10%	Birth weight	1	1%
Route (home to school)	12	11%	Depression	1	1%
School (Euclidian)	12	11%	Mental health	1	1%
School (network)	1	1%	Positive child development	1	1%
Pre-determined areas (e.g., census tract)	27	25%	Objective BMI	21	19%
Other	11	10%	Reported BMI	12	11%
			Reported school performance	1	2%
			Reported street injuries	1	1%
			Reported child pedestrian crashes	1	1%

BE measures do not add up to 108 because the measures in each study are not mutually exclusive. Euclidian stands for “as the crow flies” distance. MVPA: Moderate to Vigorous Physical Activity, PA: Physical Activity, ST: Sedentary time, PIA: Physical Inactivity, BMI: Body Mass Index.

**Table 2:** Standardised tools to measure physical activity and active travel and street environments.

**Standardised Self-Reported Tools to Measure Physical Activity and Active Travel**	**Studies**
International Physical Activity Questionnaire (IPAQ) or (IPAQ short)	2
Short Questionnaire to Assess Health-enhancing PA (SQUASH)	1
Physical Activity Questionnaire for Older Children (PAQ-C)	1
Global Physical Activity Questionnaire (GPAQ)	1
Flemish Physical Activity Questionnaire	1
Up4it Physical activity survey	1
School Physical Activity and Nutrition Environment Tool (SPAN-ET)	1
Physical Activity Location Measurement System (PALMS)	1
Various types of travel diaries	2
**Objective Tools to Measure Physical Activity and Active Travel**	
Accelerometers or GPS instruments	30
System for Observing Play and Recreation in Communities (SOPARC)	4
**Standardised Street Environment Audit Instruments (Objective)**	
Abbreviated Pedestrian Environment Data Scan (PEDS)	1
Active Neighbourhood Checklist (ANC)	1
Community Park Audit Tool (CPAT)	1
Irvine-Minnesota Inventory	2
Microscale Audit of Pedestrian Streetscapes (MAPS)	3
Neighbourhood Destination accessibility—children (NDAI-C)	1
Neighbourhood Active Living Potential (NALP)	2
Neighbourhood Destination Accessibility Index (NDAI)	1
Neighbourhood Inventory for Environmental Typology (NIfETy)	1
Neighbourhood PA environment (NPAE) windshield survey	1
Physical Activity Resource Assessment (PARA)	2
PIN3 Neighbourhood Audit Instrument	1
School Site Audits (Delaware Department of Transportation)	1
SPACES or New Zealand SPACES (NZ-SPACES)	2
Street Design Environmental Audit Tool (modified ANC)	1
**Standardised Self-Reported Street Environment Perceptions Questionnaires (Subjective)**	
Australian Children Living in Active Neighbourhoods study questionnaire (CLAN)	2
Neighbourhood Environment Walkability Scale (NEWS)	2
Neighbourhood Environment Walkability Scale—Youth Version (NEWS-Y)	6
Barriers for Active Travel to the Centre of Education (BATACE) Spanish questionnaire	1

**Table 3:** Built environment and physical activity associations.

		**PHYSICAL ACTIVITY**																					**PHYSICAL INACTIVITY**			
		**Total PA**			**Objective MVPA/PA**					**Self-Reported MVPA/PA**					**Objective Play or Park Use**			**Parent-Report Play or Park Use**					**Total Objective Sedentary Time/Physical Inactivity**			
	**BUILT ENVIRONMENT ATTRIBUTES**	**▲**	**▼**	**◂▸**	**▲**		**▼**		**◂▸**	**▲**		**▼**		**◂▸**	**▲**	**▼**	**◂▸**	**▲**		**▼**		**◂▸**	**▲**		**▼**	**◂▸**
**(i)**	**Residential Density/Use**	**9**			6	^1^, ^L4^, ^11^, ^20^, ^21^, ^24^				2	^34, 30^							1	^43^							
**(ii)**	**Land Use Mix/Diversity**	**5**	**3**		3	^5, 11, 13^	2	^10, 23^	1 ^1^			1	^30^		1 ^41^			1	^43^				1 ^35^	3	^L2, 12, 35^	2 ^1, 15^
	Food outlets, retail density, commercial activities		2				1	^21^												1	^42^					
**(iii)**	**Intersection Density or Street connectivity**	**6**	**11**		6	^L4, L6, 10,^ ^11, 23, 24^	2	^24, 8^	1 ^1^			7	^22, 25, 26, 28, 30, 32, 33^							2	^39, 43^		2 ^35, 36^	2	^12, 35^	1 ^1^
**(iv)**	**Walkability**	**4**	**2**	**1**	4	^L4, 10, 14,^ ^18^	1	^16^	1 ^1^															1	^18^	2 ^1, 15^
**(v)**	**Walking Infrastructure**	**7**	**1**		2	^5, 9, 19^	1	^16^							1 ^41^			4	^22, 38, 40, 42^	1	^39^			*1*	*^35^*	
	**Aesthetics**	**5**	1		*3*	^5,^*^11^*, *^16^*				2	^22, 34^	1	^29^													
**(vi)**	**Accessibility to Destinations (Play/Sport Destinations)**	**4**			2	^5, 23^				2	^30, 57^															
	Proximity to school	2			2	^16, 24^																		1	^37^	
	Proximity to recreation sites	3	1	1	2	^22, 24^						*1*	*^26^*	1 ^31^				1	^22^							
**(vii)**	**Availability of Parks/Public Open Spaces or Social Spaces**	**5**			5	^L4, 7, 11, 21, 24^																				
	**Green Space/Street Greenery or Natural Water**	**5**			5	*^3^* ^, 9, 13, 16, 20^																		1	^36^	
**(viii)**	**Personal Safety**	**4**			*1*	*^3^*				*1*	*^26^*							*2*	*^22, 43^*					2	^L2, 36^	
	Crime/Physical Incivilities	3	3		1	^23^	1	^7^		1	^34^	1	^25^			1 ^41^		1	^40^							
	**Traffic Safety**	**4**	**1**		1	^24^	*1*	*^10^*		*2*	*^34, 57^*							*1*	*^39^*							
	Traffic Calming	2			1	^L6^												1	^38^							
	Traffic Lights	1																1	^38^							
**(ix)**	Traffic Levels		1				1	^23^																		
	Traffic Accidents																									
	Crossing busy street		1																	1	^40^					
	Ratio of high to low-speed roads, proportion/density of main roads	1	3				3	^19, 21, 24^		1	^ ^30^ ^												1 ^36^			
	Public transport		2									1	^30^			1 ^41^										
	Pollution (air, noise)																									
	Housing (Living in a house)	**1**			1	^10^																				
	Parental Socio-economic Status	**1**			1	^10^																				
	Access to motorized vehicles at home, travel by motorized transport																						1 ^37^			
**(x)**	**Social Norms/Support, Parental Accompaniment**	**4**			4	^7, 8, 10, 24^																		1	^L2^	
	Self-efficacy																									
	Enjoyment/Satisfaction																									

▲: Positive association, ▼: Negative association, and ◂▸: no reported association. Non-superscript numbers in the cells indicate the number of associations. The superscript number corresponds to the study ID as presented in the Supplementary Material S2. L before the superscript number indicates longitudinal studies and red indicates association in the unexpected direction.

**Table 4:** Built environment and active travel or obesity associations.

		**ACTIVE TRAVEL**																			**OBESITY**			
		**Total Active Travel**			**Objective Active Travel to School**				**Self-Reported Active Travel to School**				**Parent-Reported Active Travel**				**Self-Reported Active Travel**				**Total Objective BMI**			
	**BUILT ENVIRONMENT ATTRIBUTES**	**▲**	**▼**	**◂▸**	**▲**		**▼**	**◂▸**	**▲**		**▼**	**◂▸**	**▲**		**▼**	**◂▸**	**▲**		**▼**	**◂▸**	**▲**		**▼**	**◂▸**
**(i)**	**Residential Density/Use**	**7**	2		1	^44^			5	^51, 65, L69, 53, 63^	1 ^61^		1	^72^	1 ^74^							1	91	
**(ii)**	**Land Use Mix/Diversity**	**6**	2		1	^47^			4	^52, L69, L54, 57^	2 ^52, 61^						1	^5^						
	**Food Outlets, Retail Density, Commercial Activities**	**4**							3	^51, 50, 64^			1	^74^							2 ^87, 89^	1	86	
**(iii)**	**Intersection Density or Street Connectivity**	**20**	2		3	^47, 45, 46^			10	^52, 51, 60, 65, L69, 49, 53,55, 63, 57^	1 ^52^		3	^72, 76, 73^			4	^20, 19, 5, L6^	1 ^19^			**4**	^67, 91, 84, L96^	
**(iv)**	**Walkability**	**5**							5	^55, 62, 66, L69, 71^											1 ^80^	2	^L88, 95^	
**(v)**	**Walking Infrastructure**	**7**			2	^47, 48^			3	^50, L70, 71^							2	^5, L6^			2 ^84^	2	^86, L96^	
	Aesthetics	2							1	^57^			*1*	*^76^*							1 ^34^			
**(vi)**	**Accessibility to Destinations (Play/Sport Destinations)**	**5**			1	^48^			2	^60, 57^			2	^72, 76^								2	^87, L96^	
	**Proximity to School**	**10**			3	^46, 47, 48^			7	^49, 51, 52, 61, 62, 64, 65^														
	Proximity to recreation sites																							
**(vii)**	Availability of Parks/Public Open spaces or social spaces	2							2	^L69, 71^												2	^13, L96^	
	**Green Space/Street Greenery or Natural Water**	**4**			2	^44, 48^			1	^61^			1	^76^										
**(viii)**	**Personal Safety**	**5**			1	^48^			*3*	*^65, L70, 63^*							*1*	*^L70^*						
	Crime/Physical Incivilities	1	1						1	^56^	1 ^51^										2 ^80, 89^			
	**Traffic Safety**	**4**							*2*	*^53, 57^*			*1*	*^75^*			1	^19^				2	^13, 81^	
	Traffic Calming	1		1	1	^46^		1 ^44^																
	Traffic Lights	3			1	^44^							1	^76^			1	^L6^						
**(ix)**	**Traffic Levels**		**4**				1 ^46^				2 ^55, 61^				1 ^73^						1 ^L88^			
	Traffic Accidents		1								1 ^51^													
	Crossing busy street																							
	Ratio of high to low-speed roads, proportion/density of main roads	1							1	^68^											1 ^80^			
	Public transport	1							1	^64A^											2 ^13, 84^			
	Pollution (air, noise)																							
	Housing (Living in a house)	1			1	^46^																		
	Parental Socio-economic Status																							
	Access to motorized vehicles at home, travel by motorized transport		2								2 ^L54, 65^													
**(x)**	**Social Norms/Support, Parental Accompaniment**	**6**			1	^46^			3	^L70, L54, 71^			1	^72^			1	^L70^						
	Self-efficacy	2							2	^65, 71^														
	Enjoyment/Satisfaction	3							3	^L70, 57, 71^														

▲: Positive association, ▼: Negative association, and ◂▸: no reported association. Non-superscript numbers in the cells indicate the number of associations. The superscript number corresponds to the study ID as presented in the Supplementary Material S2. L before the superscript number indicates longitudinal studies and red indicates association in the unexpected direction.

**Table 5:** Built environment categories to consider in studies of child health and examples.

Category	Example Measures
**i. Residential or population density**	Number of residents within a buffer around home, school, and/or specific route.
**ii. Street connectivity/Intersection density**	Number of intersections (e.g., total intersections, or cul-de-sacs, or 4-way intersections) within a buffer.
**iii. Land-use mix/diversity**	Proportion of different land-uses within a buffer. Entropy Index (using formulas that combine land-use classifications and the proportion of land dedicated to a specific land-use).
**iv. Walkability**	Walkability Index (using formulas that combine residential density, intersection density, and land-use mix, and other attributes such as public transit density or retail floor area ratio) within a buffer.
**v. Pedestrian infrastructure and road/street environment design**	Total length of footpaths or pavements or sidewalks (and/or width of the same) within a buffer. Network distance to nearest footpath. Parental or children’s perceived pedestrian friendliness, cleanliness, and aesthetics (e.g., interesting architecture or sights) of a street segment. Or, perceptions of the hostility of the environment (graffiti, etc.).
**vi. Accessibility or proximity to physical activity facilities**	Network distance to nearest physical activity centre, or playground, or school.
**vii. Availability or proximity to parks, public open and social spaces, and natural environments (green and blue)**	Number or total area of parks/green space/open space within a buffer. Mean NDVI (Normalised Difference Vegetation Index) within a buffer. Network distance to nearest green/blue space. Number of street trees along a street segment/route. Parent-perceived access/quality of green/blue spaces.
**viii. Safety from traffic and crime**	Parent and/or child-perceived safety from traffic and crime. Number of safety-related measures (e.g., zebra or pedestrian crossings with traffic light, slow points, speed bumps) within a buffer.
**ix. Traffic levels, presence of main roads, and characteristics of crossings**	Proportion of high-speed roads to low-speed streets within a buffer. Total length of different road types divided by the total road length within a buffer. Presence of major/arterial roads near the child’s home or school street. Density of bus stops and/or metro stations.
**x. Social support and psychosocial factors**	Reported parental or peer support for active travel to school or playing in the neighbourhood. Reported enjoyment of physical activity or active travel to school.

## Data Availability

All data supporting the reported results can be found in the Supplementary Material S2.

## Supplementary Materials

The following are available online at https://www.mdpi.com/article/10.3390/ijerph182010741/s1, Supplementary Material S1: Inclusion/exclusion criteria, Supplementary Material S2: Summary of reviewed studies presented in Table 3 and Table 4.
