# Supplementary material for: Measuring the Built Environment in Studies of Child Health—A Meta-Narrative Review of Associations
Source: Int J Environ Res Public Health. 2021 Oct 13;18(20):10741. doi: 10.3390/ijerph182010741 (PMC8535212; doi:10.3390/ijerph182010741)
Supplement: Supplementary file 1 [file ijerph-18-10741-s001.zip › Supplementary materials/Suplementary Material S1.pdf]

International Journal of
Environmental Research and Public Health

MDPI

Supplementary Material S1: Selection Criteria

References were considered to be eligible if they had the following:

- Quantitative, objective or systematic, measures of physical or spatial aspects of the street and urban built environment in the public realm. Examples of these are as follows:
    - GIS measures such as street network distance from home to school.
    - Measures gathered via built environment audits (on-site or virtual) completed by trained raters
    - Questionnaires or surveys completed by people about their perception of built environment features using a Likert scale (e.g. Healthy Streets indicators).
- Any of the three measures above used in a study involving mixed methods, such as a walk-along interview using a GIS mapped route **AND**
- Objective, observed, or self-reported measures of physical or social activities such as walking, cycling, active travel, recreation walking, playing, volunteering or park use. **OR**
- Objective or self-reported measures of physical or mental health and wellbeing:
- Health outcomes such as body mass index (BMI), number and types of traffic-related injuries, and reduction of non-communicable diseases.
- Mental health outcomes such as depression or stress.
- General wellbeing outcomes such as happiness or satisfaction. Social outcomes such as social cohesion and perceptions of safety.
- Studies measuring health outcomes and health behaviours in relation to children and young people

Exclusion criteria

- Studies located in rural areas or low- or lower middle-income countries according to the World Bank classification (available from http://data.worldbank.org/about/country-classifications).
- Studies that consider cycling but without reference to walking environments or activity AND studies purely about road collisions, or did not consider the built environment (beyond road geometry).
- Pilot studies (unless they constituted a comprehensive study in their own right) OR studies with a primary focus on methods development (including audit tools and protocols) or simulations and models OR Documents which were commentaries, dissertations or books.

Systematic reviews were excluded at this point, but were flagged for further reading in order to assess if they covered any material otherwise missed.
